# SNPs in genes encoding for IL-10, TNF-α, and NFκB p105/p50 are associated with clinical prognostic factors for patients with Hodgkin lymphoma

**DOI:** 10.1371/journal.pone.0248259

**Published:** 2021-03-08

**Authors:** Rafael Dezen Gaiolla, Marcelo Padovani de Toledo Moraes, Deilson Elgui de Oliveira

**Affiliations:** 1 Viral Carcinogenesis and Cancer Biology Research Group (ViriCan), Institute of Biotechnology (IBTEC), São Paulo State University (UNESP), Botucatu, São Paulo, Brazil; 2 Hematology, Department of Internal Medicine, Botucatu Medical School, São Paulo State University (UNESP), Botucatu, São Paulo, Brazil; 3 Department of Pathology, Botucatu Medical School, São Paulo State University (UNESP), Botucatu, São Paulo, Brazil; University of Nebraska-Lincoln, UNITED STATES

## Abstract

Classical Hodgkin lymphoma (cHL) is a B-cell-derived malignant neoplasia that has a unique histological distribution, in which the scarce malignant Hodgkin and Reed-Sternberg cells are surrounded by nonmalignant inflammatory cells. The interactions between the malignant and inflammatory cells are mediated by aberrantly produced cytokines, which play an important role in tumor immunopathogenesis. Single nucleotide polymorphisms (SNPs) in genes encoding cytokines and their regulatory proteins may influence the peripheral levels of these molecules and affect disease’s pathobiology. In this study, we evaluate SNPs in the promoter regions of the genes encoding for two key cytokines in Hodgkin lymphoma: IL-10 (SNP/p*IL10*–592, rs1800872; and SNP/p*IL10*–1082, rs1800896) and TNF-α (SNP/p*TNF* -238, rs361525; and SNP/p*TNF* -862, rs1800630), as well as an SNP in the intronic region of the NFκB1 gene (SNP/i*NFKB1*, rs1585215), an important regulator of cytokine gene expression. We then look to their possible association with clinical and laboratory features in cHL patients. Seventy-three patients with cHL are genotyped by qPCR-high resolution melting. The SNPs’ genotypes are analyzed individually for each SNP, and when more than two allelic combinations are identified, the genotypes are also divided into two groups according to proposed biological relevance. By univariate analysis, patients harboring SNP/p*TNF* -238 AG genotype more frequently have EBV-associated cHL compared to homozygous GG, whereas the presence of mediastinal disease (bulky and nonbulky) is more common in the p*IL10*–592 AC/CC group compared to the AA homozygous group. Patients with SNP/i*NFKB1* AA genotype more frequently have stage IV and extranodal disease at diagnosis. These results indicate that some SNPs’ genotypes for IL-10 and TNF-α genes are associated with prognostic parameters in cHL. For the first time, the SNP/i*NFKB1* is described in association with clinical features of the disease.

## Introduction

Hodgkin lymphoma (HL) is a B-cell-derived malignant neoplasia that accounts for approximately 10% of all human lymphomas. The disease has a unique histological appearance in which the neoplastic component—the Hodgkin and Reed-Sternberg cells (H-RS)—is scarce, and tumors are mostly formed by nonmalignant inflammatory cells [1, 2]. Cure rates for the classical form of HL (cHL) reach 90% with modern treatment protocols based on chemo and radiotherapy [3–7].

Although prognostic models based on clinical and laboratory parameters for cHL are useful in clinical practice [8–10], they may not consider the disease pathobiology, which may account for their unsatisfactory performance for treatment stratification. For long-term cHL survivors, the high frequency of treatment-related late toxicity is often observed during clinical follow-up [11, 12]; furthermore, a considerable number of cHL cases relapse, including some of those initially classified as low risk [13]. Thus, data on new putative prognostic factors to accurately predict cHL behavior and outcomes are of major interest in the clinical setting.

The interactions between H-RS and inflammatory cells are mediated by cytokines, which play an important role in cHL immunopathogenesis [14, 15]. Several cytokines and chemokines have been studied in HL, but only a few are related to clinical behavior and disease features. It is worth noting that interleukin 10 (IL-10) and tumor necrosis factor alfa (TNF-α) are of great importance because they are considered major players in the regulation of inflammatory and immune responses, both known as key phenomena in the biology of HL. For instance, H-RS cells express IL-10 [16], which suppresses cytotoxic Th1 immune response [17] and contributes to the extended life span of H-RS cells [18, 19]. They also express TNF-α [20, 21], which stimulates the phagocytic and microbicidal function of macrophages and regulates the production of several other proinflammatory cytokines [22]. Elevated circulating levels of both IL-10 and TNF-α in patients with cHL were previously associated with a more aggressive disease behavior and poorer outcomes [23–28]. In 2018, His et al. reported that the IL-10 serum levels at the end of therapy distinguished patients with shorter progression-free survival and overall survival, suggesting a potential role for this parameter in identifying patients at higher risk for relapse after the initial risk-adapted therapy [29].

Single nucleotide polymorphisms (SNPs) are genetic variations that may influence the peripheral levels of cytokines, including IL-10 and TNF-α [30–33]. The cytokine production is also modulated by intracellular signaling pathways, and even their activity is affected by SNPs in their respective genes. Notably, the nuclear factor kappa B (NFκB) pathway transcriptionally regulates numerous cytokine genes, and its abnormal activation is highly common in lymphomagenesis, including for cHL [34, 35]. The NFκB signaling is also hijacked by the Epstein-Barr virus (EBV), which is a key event in the pathogenesis of EBV-associated cHL [36].

The NFκB family of transcription factors includes the p65 (RelA), RelB, c-Rel, p50/p105, and p52/p100 proteins, which act as either homo- or heterodimers in mammalian cells [37]. Genetic variations in *NFKB1*, which encodes p50, the most common subunit for the NFκB transcription factor complex, were previously associated with increased risk for HL [38]. Other associations such as higher risk for infectious conditions [39], autoimmune diseases [40], and cancers [41, 42] have also been described.

The impact of gene polymorphisms on the pathobiology and prognosis of cHL is currently a disputed topic because of the insufficient or conflicting data published. Thus, this study is aimed at evaluating possible associations of SNPs for the promoter regions of the genes encoding for IL-10 and TNF-α, as well as one SNP within the *NFKB1* gene intronic region, identified in a series of cHL patients and non-cHL controls. The results are then evaluated considering putative associations of the SNPs’ genotypes found with histologic subtypes of cHL, EBV infection status, and well-established prognostic parameters for cHL.

## Materials and methods

### Subjects

The study retrospectively included living patients with a diagnosis of HL between January 01, 1999 and November 30, 2010 who were treated at Botucatu Medical School’s Academic Hospital. From December 1^st^ 2010 to December 31^st^ 2014, consecutive patients with HL diagnosis were included prospectively. Eligible patients were aged 16 years or older at the moment of HL diagnosis.

Baseline features at diagnosis (e.g., age, gender, stage, presence of bulky mediastinal mass, extranodal disease, number of involved nodal areas, and several laboratory parameters) were retrieved from electronic medical records for all cHL cases. The cHL disease stage was defined according to the Ann-Arbor classification [43], and treatment was defined according to cHL-standardized institutional chemotherapeutic protocols. By the time the enrolled patients were treated, positron emission tomography-computed tomography (PET-CT) was not available in our institution; thus, a conventional CT scan was used for imaging evaluation instead.

All cHL patients were classified according to the International Prognostic Score (IPS) [8] and the German Hodgkin Study Group (GHSG) risk group score [44]. The IPS takes into account seven risk factors associated with disease outcomes (e.g., progression-free survival and overall survival), each of them with similar and independent prognostic effect: male sex, age of 45 years or older, stage IV disease, serum albumin levels less than 4 g/dL, hemoglobin level less than 10.5 g/dL, leukocytosis with more than 15,000/mm^3^ white-cells count, and lymphocytopenia (lymphocytes of less than 600/mm^3^ of blood). Patients with up to two risk factors were classified as low-risk and those with three or more risk factors as high-risk cHL. The GHSG is largely used to guide treatment decisions for the best strategy, considering: Ann Arbor stage, number of involved nodal sites, erythrocyte sedimentation rate, presence of bulky mediastinal mass, and involvement of extranodal sites. According to the GHSG score, the cHL cases are classified as early disease stage with favorable prognosis, early disease stage with unfavorable prognostic features, or advanced disease stage. For the purpose of the statistical analysis in this study, the cHL cases were dichotomously divided into the group of patients with advanced disease and those with nonadvanced cHL (early stages of favorable and unfavorable disease).

For inferences regarding the frequencies and distribution of SNP genotypes, blood-donor volunteers matched to cHL patients according to age, gender, and skin color (as an ethnicity proxy) were also recruited to generate a non-cHL control group.

This study was approved by the Ethical Committee on Research at the Medical School of Botucatu, Sao Paulo State University (UNESP), SP, Brazil (Protocol #523/10), and informed consent was obtained for all subjects before enrollment.

### Histopathology, immunophenotype, and EBV infection status

All tumor specimens were reviewed by an experienced pathologist using tissue microarray slides (58/73 cases; 79,5%), produced as previously described [45], or archived stained tissue slides (15/73 cases; 20,5%). Unstained tumor sections were used for immunohistochemistry (IHC) with anti-CD45 (clone DD7/26, 1:250), anti-CD20 (clone L26, 1:700), anti-CD30 (clone Ber-H2, 1:100), anti-CD15 (clone C3D-1, 1:150), and anti-CD3 (clone 565; 1:800) antibodies. The EBV infection status was assessed by *in situ* hybridization against EBV-encoded RNA 1 (EBER-1), as reported elsewhere [46], and/or by IHC with anti-EBV latency membrane protein-1 (LMP-1) (CS1-4, 1:100). All antibodies used were manufactured by Dako Cytomation (Carpinteria, CA, USA).

### DNA extraction

Conventional venipuncture-drawn blood samples were obtained from all participants using 4mL BD Vacutainer^™^ EDTA tubes (Beckson-Dickson, Rutherford, NJ, USA). Mononuclear cells were isolated with *Histopaque*^™^ (Sigma-Aldrich, St. Louis, MO, USA) after centrifugation at 400xg for 30min. DNA extraction was performed using the *QIAamp DNA Blood Mini Kit*^™^ (Qiagen, Valencia, CA, USA) according to the manufacturer’s instructions. The DNA was eluted, quantified with the NanoVue^™^ device (GE Healthcare, Buckinghamshire, UK), and kept at -20°C until use.

### SNPs genotyping

The target genes and SNPs evaluated in this study were selected based on previous data in the literature about their putative role in cHL pathogenesis. This included SNPs in the promoter regions of the *IL10* gene (NCBI’s GeneID: 3586) at positions -592 (rs1800872; SNP/p*IL10*–592, C>A) and -1082 (rs1800896; SNP/p*IL10*–1082, A>G); in the promoter regions of the *TNF* gene (NCBI’s GeneID: 7124) at positions -238 (rs361525; SNP/p*TNF* -238, G>A) and -862 (rs1800630; SNP/p*TNF* -862, C>A); and in the intronic region of *NFKB1* gene (NCBI’s GeneID: 4790, rs1585215: SNP/i*NFKB1*, A>G). Briefly, DNA samples were normalized to 20ng/μL and amplified by real-time polymerase chain reaction (qPCR)-high resolution melting (HRM) using the Rotor-Gene^™^ 6000 equipment (Corbett Research, Sydney, Australia). qPCR primers were obtained from literature for SNPs/p*IL10*–592 and -1082 [47] and for SNPs/p*TNF* -238 and -862 [48, 49], while primers to evaluate the SNP/i*NFKB1* were designed by the authors (RDG and DEO) [50]. The oligonucleotide sequences and amplicon sizes are provided in S1 Table. qPCR assays conditions are provided in S2 Table.

The genotyping analysis was performed by comparing melting curves for the cHL case samples and controls for each SNP genotype under consideration, previously validated by conventional DNA sequencing (Sanger’s method). Both HRM and conventional melting curves were used to improve the discrimination of the SNPs genotypes, and all samples were evaluated with technical duplicates.

### Genotype-to-phenotype inferences

To subsidize the analysis of possible biological effects of the investigated SNPs in patients with cHL, we searched for public datasets that could present information about the expression levels of the genes encoding for IL-10, TNF-α, and NFκB in other cohorts of cHL cases. To the best of our efforts, by October 2020 no suitable datasets were found using the NCBI GEO (https://www.ncbi.nlm.nih.gov/sites/GDS; search query available via https://bit.ly/3lRAafY), ExSNP (http://www.exsnp.org/; last updated in 2014) [51], GWASdb v2 (http://jjwanglab.org/gwasdb; last updated in 2015) [52], CausalDb (http://mulinlab.org/causaldb) [53], and Enrichr via Harmonizone (https://maayanlab.cloud/Harmonizome/) [54]. Then, we performed a metasearch using the PhenoScanner v2 tool [55] to prospect the effects of the evaluated SNPs in the expression of their correspondent gene or related ones, querying their respective Reference SNP (rs) codes (rs1800872 for SNP/p*IL10*–592, C>A; rs1800896 for SNP/p*IL10*–1082, A>G; rs361525 for SNP/p*TNF* -238, G>A; rs1800630 for SNP/p*TNF* -862, C>A; and rs1585215 for SNP/i*NFKB1*, A>G) and investigating the significant associations found in the retrieved data.

### Statistical analysis

The group of cHL cases and its matched control group were compared for gender and age distribution of their subjects using the chi-square and Mann-Whitney tests. The Hardy-Weinberg equilibrium was assessed using the R software with SNPassoc package v1.9–2 [56].

The SNP genotypes were analyzed individually for each SNP. When more than two allelic combinations were identified, the genotypes were also divided into two groups according to proposed biological relevance described previously [32, 33, 57], as follows: for SNP/p*TNF* -238, GG vs AG/AA; for SNP/p*TNF* -862, CC vs AC/AA; for SNP/p*IL10–592*, AA vs AC/CC; for SNP/p*IL10*–1082, AA vs AG/GG; and for SNP/i*NFKB1*, AA vs AG/GG.

For the cHL patients, Chi-square or Fisher’s exact tests were used to assess the association among SNPs/p*TNF*, SNPs/p*IL10*, and SNPs/i*NFKB1* genotypes with dichotomized clinical and laboratory variables, and the risk category, based on IPS and GHSG scores. For the univariate positive associations (P≤0,05) among genotypes and clinical or laboratory variables, a multiple logistic regression model was carried out, adjusted by known relevant prognostic factors previously described in the literature (age, clinical stage, and histological subtype).

The outcome measures were progression-free survival (PFS) and overall survival (OS). According to defined international standardization [43], PFS was defined as the time from the beginning of treatment until progression or death from any cause, while overall survival was calculated from the beginning of treatment until death from any cause. Data from patients that were alive and did not progress were censored at the time of the last visit. Survival curves were generated using the Kaplan-Meier method, and differences in PFS and OS were tested for significance by log-rank test. For survival analysis, all genotypes were grouped as indicated above.

The statistical analysis was performed using the SAS Software version 9.2 (SAS Institute Inc., Cary, NC, USA). Differences were considered significant when p≤0,05.

## Results

### Features of cHL patients

Eighty patients with a confirmed diagnosis cHL were originally recruited. However, seven were excluded because they were younger than 16 years old at diagnosis (n = 5) or due to lack of paraffin-embedded tissues for histopathological review (n = 2). The main features of the 73 cHL cases evaluated are presented in Table 1.

**Table 1:** Main characteristics of cHL patients.

Features	N (%)
Gender	
Males	42 (57,5)
Females	31 (42,5)
Age	
16–44 years	60 (82,2)
≥45 years	13 (17,8)
ECOG performance ≤2	72 (98,6)
Ann-Arbor stage I/II	42 (57,5)
B symptoms	43 (58,9)
Histology subtype	
Nodular sclerosis	53 (72,6)
Mixed cellularity	10 (13,8)
Lymphocyte-rich	1 (1,3)
cHL unclassified	9 (12,3)
Bulky mediastinum mass (>10cm)	16 (21,9)
Nodal areas ≥3	33 (45,2)
Extranodal disease*	14 (19,1)
BM involvement	10 (13,7)
IPS	
0–2	26 (36)
>2	47 (64)
GHSG score	
Early favorable	4 (5)
Early unfavorable	8 (11)
Advanced	45 (62)
Missing	16 (22)
EBV infection	21/61 (34,4)

BM: bone marrow; ECOG: Eastern Cooperative Oncology Group; IPS: International prognostic score; EBV: Epstein Barr virus;

*Extranodal involvement included lung, bone, breast, palatus, adrenal gland, liver, and colon. In one patient the extranodal involvement was the only site of disease.

Fractions represent number of patients with available data.

The median age of patients was 28 years (range 16–73); 42 (57.5%) patients were male, and the male/female ratio was 1,4:1. By the time of cHL diagnosis, most of the patients (42/73; 57.5%) had disease at stage I or II, 43 (58,9%) presented with B symptoms, and all except one patient had ECOG performance status ≤ 2. After the histopathological review, 53 (72.6%) of the cHL cases were classified as nodular sclerosis and 10 (13.8%) as mixed-cellularity subtypes; the remaining cases were either lymphocyte-rich (1/73; 1,4%) or cHL unclassified (9/73; 12,3%). The EBV infection was confirmed in 21/61 (34.4%) cases. Furthermore, most cHL cases were considered as advanced disease by both IPS and GHSG scoring systems. As expected, no differences were observed regarding age and sex distribution comparing the cHL group and the control group, which comprised 73 matched non-cHL blood donor volunteers.

The majority of patients (69/73; 94.5%) was treated with doxorubicin, bleomycin, vinblastine, and dacarbazine (ABVD regimen); 2 (2.7%) patients were treated with bleomycin, etoposide, doxorubicin cyclophosphamide, vincristine, procarbazine, and prednisone (BEACOPP baseline regimen), and 2 (2.7%) with a hybrid protocol with mechlorethamine, vincristine, procarbazine, prednisone, doxorubicin, bleomycin, and vinblastine (MOPP-ABV). Involved field radiotherapy (RT) was performed as a consolidation treatment for 42 (57.5%) patients. Complete response (CR) and partial response to first-line treatment were observed in 94.5% and 3.5% of cases, respectively. Disease relapse was observed in 21.7% of patients in previous CR, and 46.1% of the relapsed patients received consolidation therapy with autologous stem-cell transplantation.

The median clinical follow-up since diagnosis was 142 months (range 16–252 months). At the time of the final analysis, 60 (82.2%) patients were alive (including 5 out of 6 transplanted). Thirteen patients (17.8%) had died: 7 (53.8%) as a result of disease progression, 3 (23%) due to infection, 1 (7.7%) due to lung cancer, and 2 (15.4%) from other reasons. The estimated PFS and OS for the studied population was 14.4 years (95% CI, 12.6–16.1) and 17.7 years (95% CI, 16.2–19.3), respectively.

### Genotype analysis and associations with patient characteristics

There was no difference in the distribution of genotypes between the cHL group and control group for all the SNPs analyzed, except for the homozygous GG of SNP/p*IL10*–1082, which was observed less frequently in the cHL group. Genotype distribution for all the investigated polymorphisms was on the Hardy–Weinberg equilibrium. SNP/p*TNF* -238 AA and SNP/i*NFKB1* GG genotypes were not found in the cHL group, but the latter was found in one subject in the control group (Table 2).

**Table 2:** Genotype distribution of SNP/p*TNF* -238 and -862, SNP/p*IL10*–592 and -1082, and SNP/i*NFKB1* in cHL patients and controls.

Polymorphism	Genotype	Patients		Controls		P
		N	%	N	%	
***TNF*-238 (rs361525)**	GG	64	87,7	68	93,2	0,25
	AG	9	12,3	5	6,8	
***TNF*-862 (rs1800630)**	AA	8	11	7	9,6	0,3
	CA	29	39,7	21	28,8	
	CC	36	49,3	45	61,6	
***IL-10*-592 (rs1800872)**	AA	8	11	8	11	0,98
	CA	34	46,6	33	45,2	
	CC	31	42,4	32	43,8	
***IL10*-1082 (rs1800896)**	AA	34	46,6	28	38,4	0,04
	GA	37	50,7	35	47,9	
	GG	2	2,7	10	13,7	
***NFKB1*** (rs1585215**)**	AA	48	65,8	43	58,9	0,49
	AG	25	34,2	29	39,7	
	GG	0	0	1	1,4	

The comparisons between SNPs’ genotypes and patients’ characteristics are detailed in S3 Table. The studied SNPs’ genotypes were not associated with specific clinical staging (I/II versus III/IV), histological subtype, or age. Univariate analysis of SNPs/p*TNF* genotypes revealed that the AG carriers of p*TNF* -238 were more frequently associated with EBV infection when compared to homozygous GG (p = 0,04; Fisher’s exact test). No other significant associations with dichotomized variables were observed with respect to SNPs/p*TNF*. In the multivariate analysis, the association of SNP/p*TNF* -238 GG with EBV was maintained when adjusted by age, histological subtype, and clinical stage (OR: 7.73; CI 95% 1.23–48.5) (P = 0.029).

Comparisons of SNPs/p*IL10* grouped genotypes of SNPs/p*IL10* with categorical variables revealed that presence of mediastinal disease (bulky and nonbulky) was more common in the p*IL10*–592 AC/CC group compared to the AA homozygous group (p = 0,002; Fisher’s exact test). In multivariate analysis, this association remains significant when adjusted for clinical stage, histological subtype, and age (OR: 21; CI 95% 2.24–197.2) (P = 0.008). No differences between these groups were observed when only bulky mediastinal mass was considered. No other significant associations were observed for the remaining studied variables and SNPs/p*IL10*.

In respect to SNP/i*NFKB1*, univariate analysis showed that the AA genotype was more frequently associated with extranodal disease when compared to AG carriers. In the multivariate analysis this association was maintained when adjusted by age, and duration of symptoms (OR: 0.23; CI 95% 0.06–0.93) (P = 0.04). When clinical stages were analyzed ungrouped, SNP/i*NFKB1* AA patients presented more frequently with stage IV disease at diagnosis (p = 0,02; chi-square test), but this finding was not sustained in the multivariate analysis (OR: 0.23; CI 95% 0.04–1.26) (P = 0.09) when adjusted by age, gender, and duration of symptoms.

In summary, SNPs/p*TNF* AG and SNP/i*NFKB1* AA genotypes were significantly associated with important known prognostic factors in HL (EBV infection and extranodal disease, respectively), both in univariate and in multivariate analysis.

Regarding clinical outcomes, no differences in PFS and OS were observed for SNP/p*TNF* -238 GG versus AG/AA; for SNP/p*TNF* -862 CC versus AC/AA; for SNP/p*IL10*–592 AA versus AC/CC; for SNP/p*IL10*–1082 AA versus AG/GG; and for SNP/i*NFKB1* AA versus AG/GG (S1–S3 Figs).

### Genotype analysis and associations with patient characteristics

To explore the possible impact of SNP/pIL10–592 and -1082, SNP/pTNF -238 and -862, and SNP/iNFKB1 on gene expression, we searched the PhenoScanner v2 database [55] to identify datasets from previous studies in which authors identified any significant biological effect documented. The results from different datasets indicate that all SNPs evaluated regulate their own gene and/or a related one (e.g., LTA, encoding TNF-β, in the case of SNP/p*TNF*s), among others identified (S4 Table).

## Discussion

Several scholars have reported that SNPs in cytokine genes may be associated with changes in the risk for developing HL [38, 58, 59], but their role in cHL biology and impact on the outcome is controversial.

Although high serum levels of TNF-α were associated with clinical features and poorer prognosis in cHL [28], data on the role of SNPs in the TNF gene in cHL are scarce. In this study, a significant association of the SNP/p*TNF* -238 AG genotype with EBV-associated cHL was found. Considering that EBV is more frequently observed in older cHL patients and MC subtype [60], the multivariate analysis was adjusted for age and histologic subtype. Previously, it was suggested that these genotypes are associated with decreased transcriptional activity because they probably affect the binding of nuclear proteins to the promoter region of the gene [32, 33]. In cHL, TNF-α is mainly produced by H-RS, and it plays a significant role in the modulation of T-cell response by recruiting Th1 cells into the tumor microenvironment and regulating the production of other proinflammatory cytokines [15]. Because Th1 response is required against EBV infection [61], it is plausible that the presence of SNPs that impair TNF-α production might adversely affect the viral clearance, allowing survival of EBV-positive H-RS malignant cells.

The prognostic value of IL-10 serum levels in HL has been previously investigated [23–26, 62]. IL-10 circulating levels seem to be influenced by SNPs/p*IL10* at positions -592 and -1082, with an impact on prognosis [31]. Here, we found that the AA genotype at SNP/p*IL10*–592 was strongly associated with less frequent mediastinal involvement (bulky and nonbulky disease). The multivariate analysis was adjusted by the histologic subtype and clinical stage, which are closely related to the pattern of nodal site involvement in cHL. Because the production of this cytokine is partially influenced by genetic background, some SNPs within the *IL10* gene may have an impact on the clinical presentation and outcomes in cHL. The presence of the A allele at either SNP/p*IL10* positions -592 or -1082 seems to be associated with lower IL-10 production compared to the G and C alleles at SNPs/p*IL10*–1082 and -592, respectively [63–65]. It has been proposed that A allele confers high ligation affinity to nuclear proteins, like PU.1 at -592 site and STAT3 at -1082 site, both of which repress IL10 gene transcription [63, 64, 66]. In this scenario, anti-inflammatory effects of IL-10 would be reduced, leading to a shift toward the production of proinflammatory cytokines, including TNF-α and IL-6, known to influence leukocyte chemoattraction (including eosinophils) and tissue redistribution, as well as lymphocyte apoptosis [67, 68]. Altogether, these mechanisms could explain some commonly observed laboratory features in cHL patients, such as anemia, eosinophilia, leukocytosis, and lymphocytopenia, particularly in advanced-stage presentation.

Different mechanisms are involved in NFκB activation in cHL, including mutations in the genes encoding for NFκB inhibitors (e.g., *NFKBIA* and *NFKBIE*) [69, 70] and genomic gains of *REL* [71]. SNP/i*NFKB1* was previously shown to influence the risk of developing HL [38], but data regarding its association with biological or clinical aspects of this disease are scarce. As far as we could verify, we are the first to investigate associations between SNP/i*NFKB1* and prognostic parameters in cHL. As previously known, introns are involved in virtually every important step of RNA processing and can therefore modify the expression level of a host gene in many ways [72]. In our study, the AA genotype of SNP/i*NFKB1*, was found to be significantly associated with advanced clinical stage and presence of extranodal disease at diagnosis compared to AG genotype by univariate analysis. Extranodal presentation is uncommon in cHL occurring in 5–16% of cases and is more frequently observed in younger ages and advanced-stage disease [73]. Likewise, the longer duration of cHL related symptoms may reflect more extensive disease, including extranodal presentation [74]. Based on this, age and duration of symptoms were included as independent variables in multivariate analysis. Extranodal cHL has also been associated with lower rates of complete remission after treatment and poorer clinical outcomes [73, 75]. Our findings suggest a possible association of this polymorphism with modulation of *NFKB1* expression, which might interfere with tumor cell growth control and inhibition of apoptosis, leading to the more aggressive behavior of cHL.

The lack of expression data for the genes encoding the cytokines TNF-α and IL10, as well as *NFKB1*, may be considered a limitation of this study. Nonetheless, it is important to consider that the transcriptional levels of these genes do not necessarily reflect directly on protein levels—the most meaningful information in a pathobiological stand point. On the other hand, although the information of serum levels of cytokines would enrich our results, they may be cumbersome to evaluate because systemic cytokine levels are typically very variable, subject to a whole range of parameters (e.g., patients’ age, gender, systemic inflammatory status, stage of the disease, ongoing treatments, etc.) that must be considered during data analysis. Besides, the tissue levels of TNF-α, IL10, and NFκB proteins are not exclusively influenced by the genetic background but also by posttranscriptional regulatory mechanisms, which contribute to the result regarding cytokines’ activities. As a matter of fact, the effect of gene polymorphisms in cytokine production is highly complex, with several variants possibly showing even combined effects [76]. Although this study did not provide new experimental or clinical data showing that the investigated SNPs may cause changes in the regulation of their respective genes, evidence supporting this idea is provided by the genotype-to-phenotype analysis we have performed with the Pheno Scanner v2 metasearch tool (S4 Table). Thus, this result strengthens our hypothesis that the associations between the increased frequency of some of the evaluated SNPs and clinical parameters in cHL cases have a biological background, even though the exact mechanisms and the net effect of these regulations in vivo remain to be elucidated.

In this study, the different genotypes of the studied SNPs were not associated with any specific histological subtype of cHL. Although the number of MC subtype in our study was lower than that previously described in Brazil, recently published data from the Brazilian Prospective Hodgkin’s Lymphoma Registry show similar results in a representative cohort of 674 patients, reporting an incidence of 13% of MC [74].

Likewise, SNPs/p*TNF 238* and *-862*, SNPs/p*IL10*–592 and -1082, and SNP/i*NFNB1* genotypes showed no association with PFS and OS, although some associations have been reported elsewhere [31, 57]. One possible explanation for these conflicting findings is the small number of patients included in the present cohort. Notably, due to the study design, most cHL cases were retrospectively included, thus inducing a potential selection bias.

The main relevant limitation of this study is the small number of patients included. Although a larger cohort of cHL would strengthen the analysis performed, this was not achieved due to the relatively small number of patients with this disease attending the tertiary academic hospital where this study was conducted. Moreover, despite our best efforts, our retrospective data collection might be associated with potential biased analysis because we were not able to include patients who had already died or had lost follow-up upon the beginning of the study.

In conclusion, we found that some genotypes for SNPs in genes encoding for TNF-α and NFκB p105/p50 were associated with known prognostic parameters for cHL. Considering that genotype—phenotype assumptions must take into account possible effects of isolated versus multiple SNPs, gene–gene, and gene–environment interactions, further analysis including haplotype combinations and gene–gene interaction models may be valuable to define more precisely the pathogenetic role of polymorphisms in cytokine-encoding genes in cHL. Additionally, multicenter clinical studies would be highly desirable to assess the applicability of the obtained results in clinical practice.

## Supporting information

S1 Table Oligonucleotide sequences used to detect SNPs/p*TNF* at positions -238 and -862, SNPs/p*IL-10* at positions –592 and -1082, and SNP/i*NFKB1* using qPCR-HRM. (DOCX)

S2 Table qPCR conditions and cycling pattern used to amplify SNPs/p*TNF* at positions -238 and -862, SNPs/p*IL-10* at positions –592 and -1082, and SNP/i*NFKB1*. (DOCX)

S3 Table Associations of SNPs/pTNF -238 and -862, SNP/pIL10–592 and -1082, and SNP/iNFKB1 with clinical features and histologic subtype of patients with cHL (N = 73). (DOCX)

S4 Table Genes regulated by the SNPs in the promoter regions of *IL10* and *TNF*, as well as in the intronic region of *NFKB1*, identified in datasets retrieved from PhenoScanner v2. (DOCX)

S1 Fig Progression-free survival and overall survival for SNPs/p*TNF*-α genotypes. (DOCX)

S2 Fig Progression-free survival and overall survival for SNPs/p*IL10* genotypes. (DOCX)

S3 Fig Progression-free survival and overall survival for SNP/i*NFKB1* genotypes. (DOCX)

S4 Fig Morphology and immunophenotype of a case of classical Hodgkin lymphoma, mixed cellularity subtype (case #80). (DOCX)

S5 Fig Morphology and immunophenotype of a case of classical Hodgkin lymphoma, nodular sclerosis subtype (case #2). (DOCX)

S6 Fig Morphology and immunophenotype of a case of classical Hodgkin lymphoma, unclassified (case #78). (DOCX)

S1 Appendix Data on gene expression regulated by the studied SNPs obtained by Pheno Scanner metasearch. (ODS)

S1 Data (XLS)
