# Supplementary material for: SNPs in genes encoding for IL-10, TNF-α, and NFκB p105/p50 are associated with clinical prognostic factors for patients with Hodgkin lymphoma
Source: PLoS One. 2021 Mar 8;16(3):e0248259. doi: 10.1371/journal.pone.0248259 (PMC7939322; doi:10.1371/journal.pone.0248259)
Supplement: S1 Table — (DOCX) [file pone.0248259.s001.docx]

**S1 Table.** Oligonucleotide sequences used to detect SNPs/p*TNF* at positions -238 and -862, SNPs/p*IL-10* at positions –592 and -1082 and SNP/i*NFKB1* using qPCR-HRM.

| **Target** | **Oligonucleotide (Sequence 5'-3')** | **Amplicon** |
| --- | --- | --- |
| SNP/p*TNF* -238 | CAGTCAGTGGCCCAGAAGAC  AGCATCAAGGATACCCCTCACA | 75 pb |
| SNP/p*TNF* -863 | ATGTAGCGGCTCTGAGGAATGGGTTAC CTACATGGCCCTGTCTTCGTTAAG | 132 pb |
| SNP/p*IL-10* -1082 | ATCCAAGACAACACTACTAA  GTGGAAGAAGTTGAAATAAC | 130 pb |
| SNP/p*IL-10* -592 | GAAGAGGTGGAAACATGTG  TAAATATCCTCAAAGTTCC | 146 pb |
| SNP/i*NFKB1* | AGCCAGCATGATAGAACTC  TCTGAGTCCTTCAGAACATG | 201 pb |
