# Supplementary material for: SNPs in genes encoding for IL-10, TNF-α, and NFκB p105/p50 are associated with clinical prognostic factors for patients with Hodgkin lymphoma
Source: PLoS One. 2021 Mar 8;16(3):e0248259. doi: 10.1371/journal.pone.0248259 (PMC7939322; doi:10.1371/journal.pone.0248259)
Supplement: S2 Table — (DOCX) [file pone.0248259.s002.docx]

**S2 Table.** qPCR conditions and cycling pattern used to amplify SNPs/p*TNF* at positions -238 and -862, SNPs/p*IL-10* at positions –592 and -1082 and SNP/i*NFKB1*.

| **Reaction** | **Reaction Composition** | **Cycling Pattern** |
| --- | --- | --- |
| **SNP/p*TNF* -238**  **G>A** | Buffer (Tris-Cl 28 mM pH 8.4 / KCl 70mM) 1X, 2.0mM MgCl_2_, 0.2mM each dNTP, 1.25U DNA polymerase (Taq), Eva Green 1X and 0.2μM of each primer. | 94°C – 10min(1x);  94°C–30s, 58°C–20s, 72°C–20s (40x) |
| **SNP/p*TNF* -862**  **C>A** | Buffer (Tris-Cl 28mM pH 8.4 / KCl 70 mM) 1X, 2.5mM MgCl_2_, 0.2mM each dNTP, 1.25U DNA polymerase (Taq) Eva Green 1X and 0.15μM of each primer. | 94°C–5min(1x);  94°C–20s, 58°C–20s 72°C–20s (40x) |
| **SNP/p*IL-10* -592**  **C>A** | Buffer (Tris-Cl 28 mM pH 8.4 / KCl 70mM) 1X, 2.0mM MgCl_2_, 0.2mM each dNTP, 1.25U DNA polymerase (Taq), Eva Green 1X and 0.3μM of each primer. | 94°C – 5min(1x);  94°C–60s, 55°C–60s, 72°C–60s (40x) |
| **SNP/p*IL-10* -1082**  **A>G** | Buffer (Tris-Cl 28mM pH 8.4 / KCl 70 mM) 1X, 3.0mM MgCl_2_, 0.2mM each dNTP, 1.25U DNA polymerase (Taq) Eva Green 1X and 0.4μM of each primer. | 94°C–5min(1x);  94°C–60s, 55°C–60s 72°C–60s (40x) |
| **SNP/i*NFKB1***  **A>G** | Buffer (Tris-Cl 28mM pH 8.4 / KCl 70 mM) 1X, 2.5mM MgCl_2_, 0.2mM each dNTP, 1.25U DNA polymerase (Taq) Eva Green 1X and 0.25μM of each primer. | 94°C–5min(1x);  94°C–30s, 55°C–20s 72°C–30s (40x) |
