# Supplementary material for: SNPs in genes encoding for IL-10, TNF-α, and NFκB p105/p50 are associated with clinical prognostic factors for patients with Hodgkin lymphoma
Source: PLoS One. 2021 Mar 8;16(3):e0248259. doi: 10.1371/journal.pone.0248259 (PMC7939322; doi:10.1371/journal.pone.0248259)
Supplement: S3 Table — (DOCX) [file pone.0248259.s003.docx]

**S3 Table**. Associations of SNPs/p*TNF* -238 and -862, SNP/p*IL10* -592 and -1082, and SNP/i*NFKB1* with clinical features and histologic subtype of patients with cHL (N=73)

| **Features (n)** | **Patients**  **(%)** | ***TNF* -238** | | **P** | ***TNF* -862** | | **P** | ***IL10* -592** | | **P** | ***IL10* -1082** | | **P** | ***NFKB1*** | | **P** |
| --- | --- | --- | --- | --- | --- | --- | --- | --- | --- | --- | --- | --- | --- | --- | --- | --- |
|  |  | ***GG*** | ***AG*** |  | ***CC*** | ***AC/AA*** |  | ***AA*** | ***AC/CC*** |  | ***AA*** | ***AG/GG*** |  | ***AA*** | ***AG*** |  |
| **Age (73)** |  |  |  |  |  |  |  |  |  |  |  |  |  |  |  |  |
| 16-44 years | 60 (82.2) | 54 (90) | 6 (10) | 0.19 | 28 (46.7) | 32 (53.3) | 0.33 | 7 (11.7) | 53 (88.3) | 1.0 | 29 (48.3) | 31 (51.7) | 0.51 | 39 (65) | 21 (35) | 0.77 |
| ≥ 45 years | 13 (17.8) | 10 (76.9) | 3 (23.1) |  | 8 (61.5) | 5 (38.5) |  | 1 (7.7) | 12 (92.3) |  | 5 (38.5) | 8 (61.5) |  | 9 (69.2) | 4 (30.8) |  |
| **Gender (73)** |  |  |  |  |  |  |  |  |  |  |  |  |  |  |  |  |
| Male | 42 (57.5) | 36 (85.7) | 6 (14.3) | 0.72 | 21 (50) | 21 (50) | 0.92 | 6 (14.3) | 36 (85.7) | 0.45 | 20 (47.6) | 22 (52.4) | 0.83 | 25 (59.5) | 17 (40.5) | 0.19 |
| Female | 31 (42.5) | 28 (90.3) | 3 (9.7) |  | 15 (48.4) | 16 (51.6) |  | 2 (6.5) | 29 (93.5) |  | 14 (45.2) | 17 (54.9) |  | 23 (74.2) | 8 (25.8) |  |
| **Ann-Arbor stage (73)** |  |  |  |  |  |  |  |  |  |  |  |  |  |  |  |  |
| I - II | 42 (57.5) | 36 (85.7) | 6 (14.3) | 0.72 | 20 (47.6) | 22 (52,4) | 0.73 | 3 (7.1) | 39 (92.9) | 0.27 | 18 (42.9) | 24 (57.1) | 0.45 | 28 (66.7) | 14 (33.3) | 0.84 |
| III - IV | 31 (42.5) | 28 (90.3) | 3 (9.7) |  | 16 (51.6) | 15 (48,4) |  | 5 (16.1) | 26 (83.9) |  | 16 (51.6) | 15 (48.4) |  | 20 (64.5) | 11 (35.5) |  |
| **Histology (73)** |  |  |  |  |  |  |  |  |  |  |  |  |  |  |  |  |
| Nodular sclerosis | 53 (72.6) | 47 (88.7) | 6 (11.3) | 0.86 | 26 (49.1) | 27 (50.9) | 0.77 | 7 (13.2) | 46 (86.8) | 0.67 | 27 (50.9) | 26 (49.1) | 0.34 | 31 (58.5) | 22 (41.5) | 0.18 |
| Mixed cellularity | 10 (13.8) | 8 (80) | 2 (20) |  | 5 (50) | 5 (50) |  | 1 (10) | 9 (90) |  | 3 (30) | 7 (70) |  | 8 (80) | 2 (20) |  |
| Lymphocyte-rich | 1 (1.3) | 1 (100) | 0 |  | 0 | 1 (100) |  | 0 | 1 (100) |  | 1 (100) | 0 |  | 1 (100) | 0 |  |
| cHL unclassified | 9 (12.3) | 8 (88.9) | 1 (11.1) |  | 5 (55.6) | 4 (44.4) |  | 0 | 9 (100) |  | 3 (33.3) | 6 (66.7) |  | 8 (88.9) | 1 (11.1) |  |
| **Bulky mediastinal mass (73)** |  |  |  |  |  |  |  |  |  |  |  |  |  |  |  |  |
| Yes | 16 (21.9) | 14 (87.5) | 2 (12.5) | 1.0 | 8 (50) | 8 (50) | 0.95 | 1 (6.3) | 15 (93.7) | 0.67 | 9 (56.2) | 7 (43.8) | 0.38 | 9 (56.2) | 7 (43.8) | 0.36 |
| No | 57 (78.1) | 50 (87.7) | 7 (12.3) |  | 28 (49.1) | 29 (50.9) |  | 7 (12.3) | 50 (87.7) |  | 25 (43.9) | 32 (56.1) |  | 39 (68.4) | 18 (31.6) |  |
| **Number of nodal areas (73)** |  |  |  |  |  |  |  |  |  |  |  |  |  |  |  |  |
| ≥ 3 | 33 (45.2) | 31 (93.9) | 2 (6.1) | 0.17 | 18 (55.5) | 15 (45.5) | 0.41 | 3 (9.1) | 30 (90.9) | 0.72 | 16 (48.5) | 17 (51.5) | 0.76 | 20 (60.6) | 13 (39.4) | 0.39 |
| < 3 | 40 (54.8) | 33 (82.5) | 7 (17.5) |  | 18 (45) | 22 (55) |  | 5 (12.5) | 35 (87.5) |  | 18 (45) | 22 (55) |  | 28 (70) | 12 (30) |  |
| **Extranodal disease (73)** |  |  |  |  |  |  |  |  |  |  |  |  |  |  |  |  |
| Present | 22 (30.1) | 19 (86.4) | 3 (13.6) | 1.0 | 13 (59.1) | 9 (40.9) | 0.27 | 1 (4.5) | 21 (95.5) | 0.42 | 9 (40.9) | 13 (59.1) | 0.52 | 18 (81.8) | 4 (18.2) | **0.05** |
| Absent | 51 (69.9) | 45 (88.2) | 6 (11.8) |  | 23 (45.1) | 28 (54.9) |  | 7 (13.7) | 44 (86.3) |  | 25 (49) | 26 (51) |  | 30 (58.8) | 21 (41.2) |  |
| **EBV infection (61)** |  |  |  |  |  |  |  |  |  |  |  |  |  |  |  |  |
| Present | 21 (34.4) | 16 (76.2) | 5 (23.8) | **0.04** | 10 (47.6) | 11 (52.4) | 0.71 | 2 (9.5) | 19 (90.5) | 0.72 | 8 (38.1) | 13 (61.9) | 0.60 | 13 (61.9) | 8 (38.1) | 0.66 |
| Absent | 40 (65.6) | 38 (95) | 2 (5) |  | 21 (52.5) | 19 (47.5) |  | 5 (12.5) | 35 (87.5) |  | 18 (45) | 22 (55) |  | 27 (67.5) | 13 (32.5) |  |
| **Features (n)** | **Patients**  **(%)** | ***TNF* -238** | | **P** | ***TNF* -862** | | **P** | ***IL10* -592** | | **P** | ***IL10* -1082** | | **P** | ***NFKB1*** | | **P** |
|  |  | ***GG*** | ***AG*** |  | ***CC*** | ***AC/AA*** |  | ***AA*** | ***AC/CC*** |  | ***AA*** | ***AG/GG*** |  | ***AA*** | ***AG*** |  |
| **IPS (73)** |  |  |  |  |  |  |  |  |  |  |  |  |  |  |  |  |
| Low risk | 47 (64) | 41 (64) | 6 (66.6) | 1.0 | 12 (46.8) | 25 (53.2) | 0.74 | 5 (10.6) | 42 (89.4) | 1.0 | 22 (46.8) | 25 (53.2) | 0.85 | 31 (64.5) | 16 (64) | 0.83 |
| High risk | 26 (36) | 23 (36) | 3 (33.3) |  | 14 (53.8) | 12 (46.1) |  | 3 (11.5) | 23 (88.5) |  | 12 (46.1) | 14 (53.8) |  | 17 (35.4) | 9 (36) |  |
| **GHSG (57)** |  |  |  |  |  |  |  |  |  |  |  |  |  |  |  |  |
| Non-advanced | 12 (21) | 1 (16.6) | 11 (21.6) | 1.0 | 7 (58.3) | 5 (41.7) | 0.9 | 2 (16.7) | 10 (83.3) | 0.63 | 4 (33.3) | 8 (66.7) | 0.36 | 9 (75) | 3 (25) | 0.5 |
| Advanced | 45 (79) | 5 (83.3) | 40 (78.4) |  | 23 (51.1) | 22 (48.9) |  | 5 (11.1) | 40 (88.9) |  | 24 (53.3) | 21 (46.7) |  | 27 (60) | 18 (40) |  |

*cHL = classical Hodgkin lymphoma; EBV = Esptein-Barr virus; IPS = international prognostic score; GHSG = German Hodgkin Study Group.
