# Supplementary material for: SNPs in genes encoding for IL-10, TNF-α, and NFκB p105/p50 are associated with clinical prognostic factors for patients with Hodgkin lymphoma
Source: PLoS One. 2021 Mar 8;16(3):e0248259. doi: 10.1371/journal.pone.0248259 (PMC7939322; doi:10.1371/journal.pone.0248259)
Supplement: S4 Table — (DOCX) [file pone.0248259.s004.docx]

**S4 Table**. Genes regulated by the SNPs in the promoter regions of *IL10* and *TNF*, as well as in the intronic region of *NFKB1*, identified in datasets retrieved from PhenoScanner v2 ([http://www.phenoscanner.medschl.cam.ac.uk](http://www.phenoscanner.medschl.cam.ac.uk/))

| **SNP evaluated** | **URL to PS^1^ results** | **Regulated genes identified^2^** |
| --- | --- | --- |
| [SNP/pIL10 -592, C>A](http://www.phenoscanner.medschl.cam.ac.uk/?query=rs1800872&catalogue=eQTL&p=1e-5&proxies=None&r2=0.8&build=37) ([rs1800872](http://www.phenoscanner.medschl.cam.ac.uk/?query=rs1800872&catalogue=eQTL&p=1e-5&proxies=None&r2=0.8&build=37)) | https://bit.ly/352Lyip | *FAIM3,* ***IL10,*** *IL19, IL24* |
| [SNP/pIL10 -1082, A>G rs1800896](http://www.phenoscanner.medschl.cam.ac.uk/?query=rs1800896&catalogue=eQTL&p=1e-5&proxies=None&r2=0.8&build=37) | https://bit.ly/2H6dtpB | *C4BPB, CD55,* ***IL10,*** *IL19* |
| [SNP/pTNF -238, G>A rs361525](http://www.phenoscanner.medschl.cam.ac.uk/?query=rs361525&catalogue=eQTL&p=1e-5&proxies=None&r2=0.8&build=37) | https://bit.ly/2IzZrwN | *DDR1, DOM3Z, FKBPL, HLA-B, HLA-C, HLA-DQA2, LOC100509457, LOC100507718, HLA-DQA1, HLA-DRB5, HLA-DRB6, HLA-S, IER3, LINC00243, LST1,* ***LTA,*** *MDC1, MICA, MICB, MRPS18B, PSORS1C2, PSORS1C3, SLC44A4, TNXA****,*** *ZBTB12* |
| [SNP/pTNF -862, C>A rs1800630](http://www.phenoscanner.medschl.cam.ac.uk/?query=rs1800630&catalogue=eQTL&p=1e-5&proxies=None&r2=0.8&build=37) | https://bit.ly/2HbHNPq | *AIF1, ATP6V1G2, ATP6V1G2, SNORD84, DDX39B, BAT1, C4A, C4B, C4A, LOC100293534, CSNK2B, DDAH2, DDX39B, DDX39B, ATP6V1G2-DDX39B, DDX39B, MCCD1, ATP6V1G2-DDX39B, DHFRP2, DOM3Z, HCG27, HCP5, HLA-C, HLA-DRB1, HLA-DRB6, LOC100507714, LOC100507709, HLA-DRB5, HLA-DRB5, HLA-DRB1, LOC100507714, LOC100507709, HLA-DRB6, HLA-F-AS1, HLA-S, LINC00243, LST1,* ***LTA,*** *LY6G5B, MICA, MICB, POU5F1, PRRC2A, PSORS1C3, SLC44A4, TCF19,* ***TNF,*** *TNXB****,*** *TUBB, VARS2, XXbac-BPG299F13.16* |
| [SNP/iNFKB1, A>G rs1585215](http://www.phenoscanner.medschl.cam.ac.uk/?query=rs1585215&catalogue=eQTL&p=1e-5&proxies=None&r2=0.8&build=37) | https://bit.ly/2H73pfV | *APLF, SPTLC1, ARAP3, CISD2, FBXW7, FLVCR1, GHRL, IL2RG, CXorf65, KRT8P46, LRRC37A15P, MANBA, NELL2, MAFG, LOC100653255, LOC100653018,* ***NFKB1****, OPTN, REPS2, RP11-10L12.1, RP11-10L12.2, RP11-10L12.4, TTC25, UBE2D3* |

1.PS: PhenoScaner v2 2.The specific genes for each of the SNPs evaluated are underlined (*IL1*, *TNF*, *NFKB1*), as well as LTA, which encodes the TNF-beta cytokine.
