# Supplementary material for: SNPs in genes encoding for IL-10, TNF-α, and NFκB p105/p50 are associated with clinical prognostic factors for patients with Hodgkin lymphoma
Source: PLoS One. 2021 Mar 8;16(3):e0248259. doi: 10.1371/journal.pone.0248259 (PMC7939322; doi:10.1371/journal.pone.0248259)
Supplement: S1 Fig — (DOCX) [file pone.0248259.s005.docx]

**S1 Fig**. **Progression-free survival and overall survival for SNPs/p*TNF-α* genotypes.**

Kaplan-Meier estimates of (A) progression-free survival and (B) overall survival in patients with *TNF-α* -238 GG *versus* GA genotypes; and (C) progression-free survival and (D) overall survival in patients with *TNF-α* -862 CC *versus* AA/AC genotypes.
