# Supplementary material for: SNPs in genes encoding for IL-10, TNF-α, and NFκB p105/p50 are associated with clinical prognostic factors for patients with Hodgkin lymphoma
Source: PLoS One. 2021 Mar 8;16(3):e0248259. doi: 10.1371/journal.pone.0248259 (PMC7939322; doi:10.1371/journal.pone.0248259)
Supplement: S2 Fig — (DOCX) [file pone.0248259.s006.docx]

**S2 Fig**. **Progression-free survival and overall survival for SNPs/p*IL10* genotypes.**

Kaplan-Meier estimates of (A) progression-free survival and (B) overall survival in patients with *IL-10* -592 AA versus AC/CC genotypes; and (C) progression-free survival and (D) overall survival in patients with *IL10* -1082 AA *versus* AG/GG genotypes.
