# Supplementary material for: SNPs in genes encoding for IL-10, TNF-α, and NFκB p105/p50 are associated with clinical prognostic factors for patients with Hodgkin lymphoma
Source: PLoS One. 2021 Mar 8;16(3):e0248259. doi: 10.1371/journal.pone.0248259 (PMC7939322; doi:10.1371/journal.pone.0248259)
Supplement: S3 Fig — (DOCX) [file pone.0248259.s007.docx]

**S3 Fig**. **Progression-free survival and overall survival for SNP/i*NFKB1* genotypes.**

Kaplan-Meier estimates of (A) progression-free survival and (B) overall survival in patients with *NFKB1* AA *versus* AG genotypes.
