# Supplementary material for: SNPs in genes encoding for IL-10, TNF-α, and NFκB p105/p50 are associated with clinical prognostic factors for patients with Hodgkin lymphoma
Source: PLoS One. 2021 Mar 8;16(3):e0248259. doi: 10.1371/journal.pone.0248259 (PMC7939322; doi:10.1371/journal.pone.0248259)
Supplement: S4 Fig — (DOCX) [file pone.0248259.s008.docx]

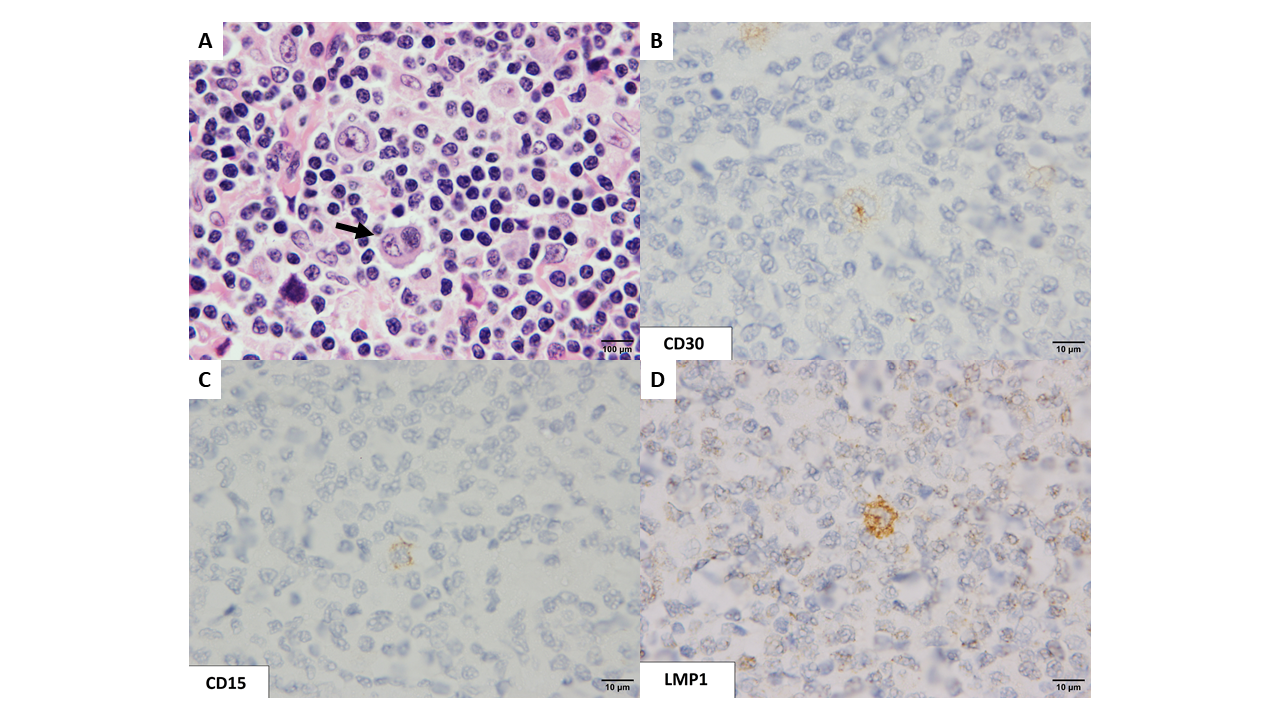
**S4 Fig. Morphology and immunophenotype of a case of classical Hodgkin lymphoma, mixed cellularity subtype (case #80).** (A) Hematoxilin-Eosin staining with a large Hodgkin/Reed-Sternberg (HRS) cell in the center (arrow). (B) Expression of CD30 showing the typical membrane and Golgi pattern of CD30 staining in a HRS cell. (C) Expression of CD15. (D) Expression of LMP1 in a HRS cell.
