# Supplementary material for: SNPs in genes encoding for IL-10, TNF-α, and NFκB p105/p50 are associated with clinical prognostic factors for patients with Hodgkin lymphoma
Source: PLoS One. 2021 Mar 8;16(3):e0248259. doi: 10.1371/journal.pone.0248259 (PMC7939322; doi:10.1371/journal.pone.0248259)
Supplement: S5 Fig — (DOCX) [file pone.0248259.s009.docx]

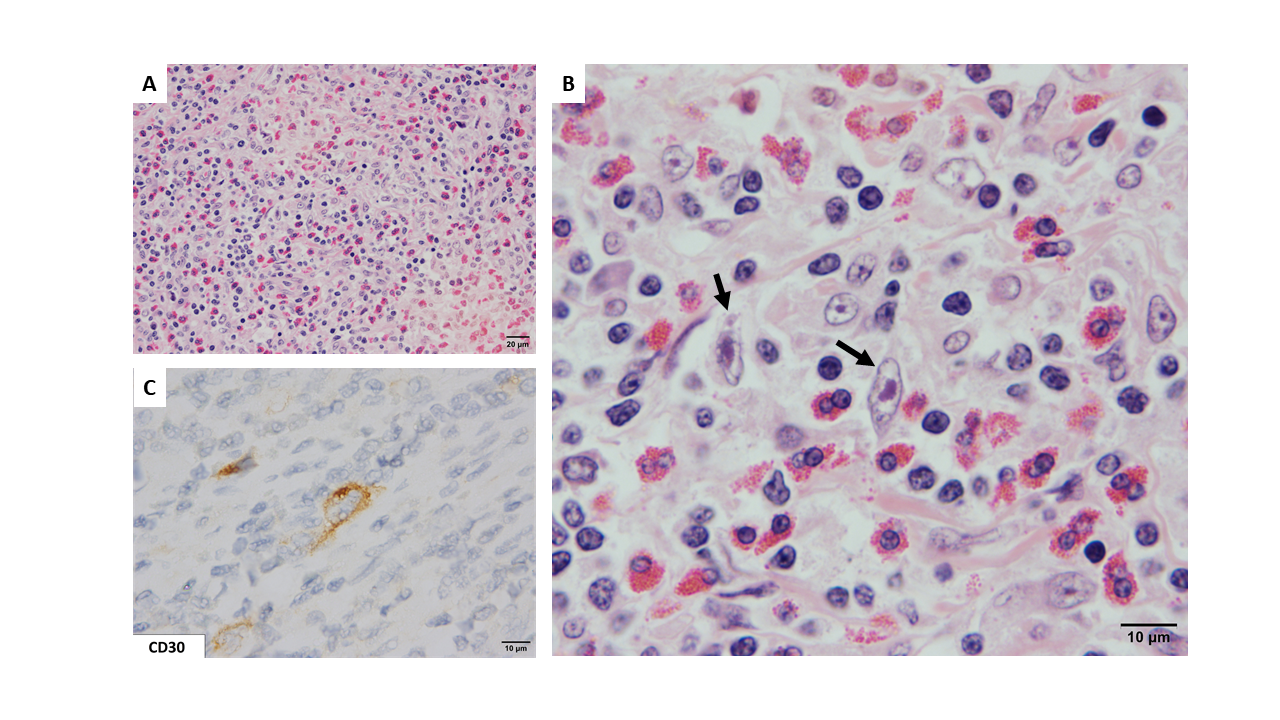


**S5 Fig. Morphology and immunophenotype of a case of classical Hodgkin lymphoma, nodular sclerosis subtype (case #2).** (A) Hematoxilin-eosin staining, low power field, with many Hodgkin/Reed-Sternberg (HRS) cells surrounded by an inflammatory background with marked eosinophilia. (B) Hematoxilin-eosin staining, high power field. The arrows show two HRS cells. (C) Expression of CD30 showing the typical membrane and Golgi pattern of CD30 staining in a HRS cell.
