# Supplementary material for: SNPs in genes encoding for IL-10, TNF-α, and NFκB p105/p50 are associated with clinical prognostic factors for patients with Hodgkin lymphoma
Source: PLoS One. 2021 Mar 8;16(3):e0248259. doi: 10.1371/journal.pone.0248259 (PMC7939322; doi:10.1371/journal.pone.0248259)
Supplement: S6 Fig — (DOCX) [file pone.0248259.s010.docx]

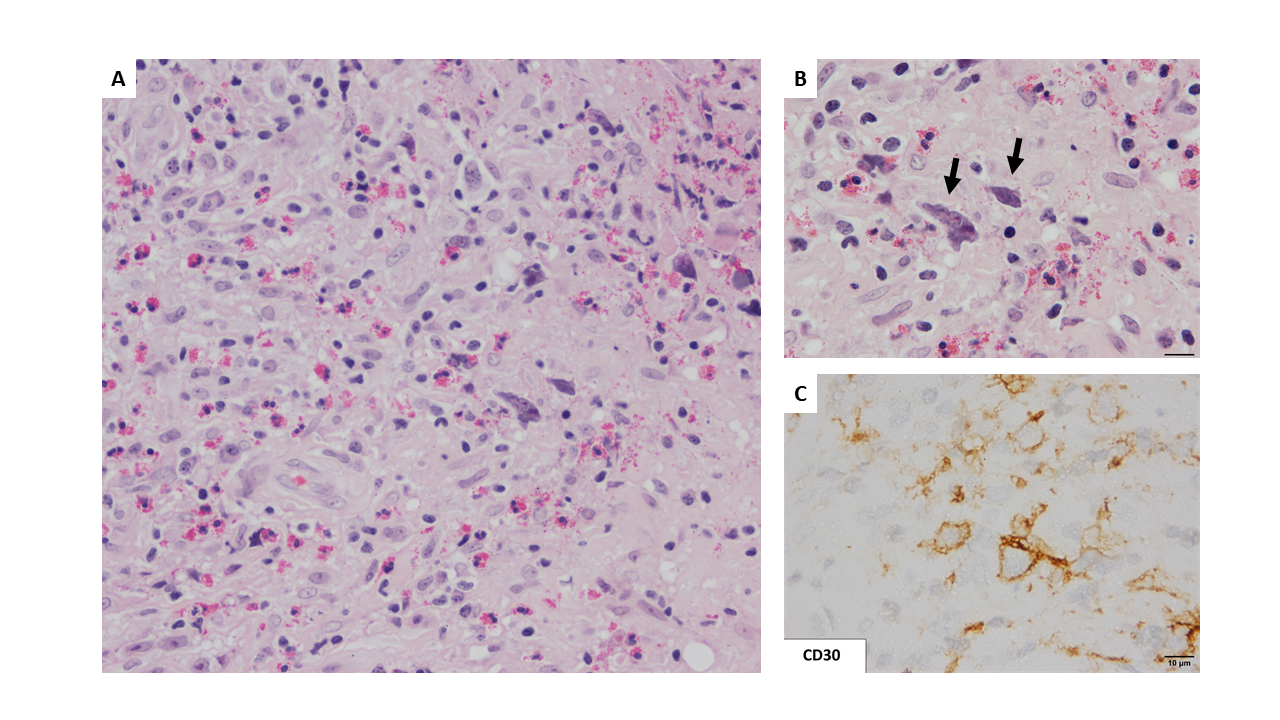


**S6 Fig. Morphology and immunophenotype of a case of classical Hodgkin lymphoma, unclassified (case #78).** This biopsy was obtained from a spine lesion. (A) Hematoxilin-eosin staining, low power field, with some Hodgkin/Reed-Sternberg (HRS) cells surrounded by an inflammatory background with eosinophilia. (B) Hematoxilin-eosin staining, high power field. Sparce HRS and apoptotic cells (arrows) are observed. (C) Expression of CD30 HRS cells.
